# *Lactiplantibacillus plantarum* HY7718 Modulates Gut–Kidney Axis-Associated Inflammation, Gastrointestinal Dysfunction, and Gut Microbiota in Adenine-Induced Chronic Kidney Disease Mice

**DOI:** 10.3390/ijms27104348

**Published:** 2026-05-13

**Authors:** Hyeonji Kim, Ji-Woong Jeong, Joo-Yun Kim, Jae-Jung Shim, Jae-Hwan Lee

**Affiliations:** R&BD Center, hy Co., Ltd., 22, Giheungdanji-ro 24beon-gil, Giheung-gu, Yongin-si 17086, Republic of Korea; skyatk94@gmail.com (H.K.); woongshow@hy.co.kr (J.-W.J.); jjshim@hy.co.kr (J.-J.S.); jaehwan@hy.co.kr (J.-H.L.)

**Keywords:** chronic kidney disease, gut–kidney axis, *Lactiplantibacillus plantarum*, intestinal inflammation, gut microbiota

## Abstract

Chronic kidney disease (CKD), a progressive disorder leading to renal dysfunction, remains a significant global health issue. This study investigated whether *Lactiplantibacillus plantarum* HY7718 modulates gut–kidney axis-associated inflammatory, gastrointestinal, and microbial alterations in a mouse model of adenine-induced chronic kidney disease. We examined fibrosis- and inflammation-related gene expression in mouse tissues and analyzed the gut microbiota via next-generation sequencing. HY7718 supplementation was associated with reduced expression of genes related to renal fibrosis (*Col1a1*, *Acta2*) and vascular inflammation (*Icam-1*, *Vcam-1*). Further, HY7718 suppressed intestinal inflammatory responses, including downregulation of pro-inflammatory cytokines (*Tnf*, *Il-1β*, *Il-6*) and TLR4/MyD88/NF-κB signaling pathway genes in the colon tissues. Gastrointestinal function was also improved, with significant upregulation of gastric motility-related genes and increased digestive enzyme activity. The gut microbiota composition was altered by HY7718, with reduced abundance of pro-inflammatory taxa such as *Mucispirillum* and *Deferribacterota*, whereas beneficial genera like *Lactiplantibacillus* were enriched. These microbial shifts were associated with reduced intestinal inflammatory and renal fibrosis-related markers. Overall, the findings indicate that HY7718 supplementation modulates gut–kidney axis-associated inflammatory, gastrointestinal, and microbial alterations in adenine-induced CKD mice and supports further investigation of this strain in CKD-related settings.

## 1. Introduction

Chronic kidney disease (CKD), a progressive condition characterized by the gradual loss of renal function, represents a major global health concern [1]. The global prevalence of CKD has steadily increased and is associated with substantial morbidity and mortality [2]. CKD progression is driven by multiple pathological processes, including persistent inflammation, oxidative stress, and structural remodeling of the renal tissue [3]. In particular, tubulointerstitial fibrosis is a key pathological hallmark contributing to irreversible renal damage and functional decline [4]. Further, endothelial dysfunction and vascular inflammation have been increasingly implicated in renal injury progression through mechanisms such as leukocyte adhesion, microvascular impairment, and tissue hypoxia [5]. Despite advances in clinical management, effective strategies for preventing or slowing CKD progression remain limited, highlighting the need for novel therapeutic approaches targeting the mechanisms underlying disease progression.

The gut–kidney axis, encompassing interactions between the intestinal environment and renal function, has attracted increasing attention in the context of CKD progression [6]. Notably, impaired renal function can alter the intestinal microenvironment and promote inflammatory responses in the gut. In particular, intestinal inflammation has been recognized as an important factor contributing to systemic inflammation and further aggravation of renal injury [7]. Activation of inflammatory signaling pathways such as the TLR4–MyD88–NF-κB pathway can stimulate the production of pro-inflammatory cytokines and amplify inflammatory responses in intestinal tissues [7,8]. Further, CKD has been associated with gut dysbiosis, which may further disturb intestinal homeostasis [9]. These alterations may promote the generation of microbial-derived metabolites and inflammatory mediators that enter systemic circulation, thereby contributing to chronic inflammation. Such systemic inflammatory responses accelerate renal injury and CKD progression [7,10,11]. Collectively, these findings highlight the critical role of the gut–kidney axis in linking intestinal inflammation and microbial imbalance to CKD.

Beyond intestinal inflammation and microbial imbalance, gastrointestinal dysfunction (GI dysfunction) has been increasingly recognized in patients with CKD. Patients with CKD frequently exhibit altered gastrointestinal motility and experience symptoms such as dyspepsia and nausea, which impairs normal digestive function [12,13]. Gastric motility disturbances may affect gastrointestinal smooth muscle activity and disrupt the normal transit of luminal contents [14]. Collectively, these gastrointestinal alterations may further compromise intestinal homeostasis and potentially contribute to CKD progression through the gut–kidney axis.

As the role of the gut–kidney axis in CKD progression has gained increasing recognition, probiotics, as potential modulators of intestinal homeostasis, have attracted considerable interest [15]. As proposed by the FAO/WHO in 2001, probiotics are defined as “live microorganisms that, when administered in adequate amounts, confer a health benefit on the host” [16]. Recent studies have reported that probiotics exert a range of beneficial effects on the host, including improvement of microbial balance, attenuation of inflammatory responses, and modulation of host metabolic pathways [17]. Among probiotic species, *Lactiplantibacillus plantarum* (*L. plantarum*) has been extensively investigated because of its ability to survive in the gastrointestinal environment and exert immunomodulatory and anti-inflammatory effects [18]. These characteristics suggest that *L. plantarum* may contribute toward intestinal homeostasis maintenance and potentially influence disease progression through the gut–kidney axis.

*Lactiplantibacillus plantarum* HY7718, isolated from fermented squid, has been reported to exert ameliorative effects in experimental CKD models [19]. HY7718 supplementation attenuated renal dysfunction and kidney injury, accompanied by reducing renal inflammation and apoptosis-related markers. However, whether these beneficial effects are associated with modulation of the gut–kidney axis remains to be fully elucidated. Therefore, in this study, we investigated whether HY7718 could attenuate adenine-induced CKD by modulating intestinal inflammation, gastrointestinal dysfunction, and gut microbial composition. Specifically, we examined renal fibrosis- and endothelial dysfunction-related gene expression, colonic inflammatory signaling, gastric motility-related genes, digestive enzyme activity, and gut microbiota profiles in adenine-induced CKD mice.

## 2. Results

### 2.1. Effect of HY7718 on Gene Expression Related to Renal Fibrosis and Endothelial Dysfunction and on Histological Alterations

**Figure 1:**
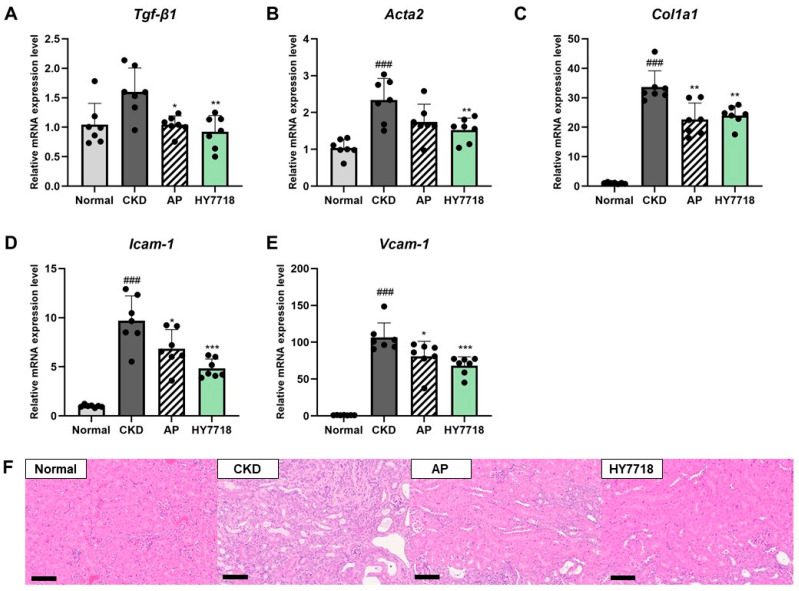
Effect of HY7718 on gene expression related to renal fibrosis and endothelial dysfunction and on histological alterations in mice with adenine-induced CKD. Relative mRNA expression levels of fibrosis-related genes (**A**) *Tgf-β1*, (**B**) *Acta2* and (**C**) *Col1a1* and endothelial dysfunction-related genes (**D**) *Icam-1*, and (**E**) *Vcam-1* in kidney tissues. (**F**) Hematoxylin and eosin (H&E) staining of kidney tissues (magnification, ×200; scale bar = 100 μm). Data are presented as the mean ± standard deviation. Statistical significance was determined by one-way ANOVA followed by Tukey’s post hoc test. ^###^ *p* < 0.001, compared with the Normal group; * *p* < 0.05, ** *p* < 0.01 and *** *p* < 0.001, compared with the CKD group. *Tgf-β1*, Transforming Growth Factor Beta 1; *Acta2*, actin alpha 2 (smooth muscle); *Col1a1*, Collagen type I alpha 1 chain; *Icam-1*, Intercellular Adhesion Molecule 1; *Vcam-1*, Vascular Cell Adhesion Molecule 1; CKD, adenine-induced CKD group; AP, allopurinol; HY7718, *L. plantarum* HY7718.

To evaluate the effects of HY7718 on renal fibrosis and endothelial dysfunction, the expression levels of fibrosis-related genes (*Tgf-β1*, *Acta2* and *Col1a1*) and endothelial dysfunction markers (*Icam-1* and *Vcam-1*) were analyzed in kidney tissues. As shown in Figure 1A–C, the expression levels of *Acta2* and *Col1a1* were significantly elevated in the CKD group compared with those in the Normal group (*p* < 0.001), whereas *Tgf-β1* expression showed an increasing trend but did not reach statistical significance. HY7718 administration significantly reduced the expression levels of *Tgf-β1* and *Acta2* and *Col1a1* (*p* < 0.01). Treatment with allopurinol (AP) also decreased the expression level of *Tgf-β1*, *Acta2*, and *Col1a1*, showing effects comparable with those observed in the HY7718-treated group. AP treatment significantly reduced the expression levels of *Tgf-β1* and *Col1a1* compared with those in the CKD group; however, although *Acta2* expression levels tended to decrease, the change was not statistically significant.

As shown in Figure 1D,E, the endothelial dysfunction-related genes *Icam-1* and *Vcam-1* showed markedly increased expression levels in the CKD group compared with those in the Normal group (*p* < 0.001). Administration of HY7718 significantly reduced the expression levels of both genes (*p* < 0.001), and AP treatment also significantly decreased the *Icam-1* and *Vcam-1* expression levels (*p* < 0.05). Overall, the reduction in the expression levels of these genes was more pronounced in the HY7718 group.

In Figure 1F, hematoxylin and eosin (H&E)-stained sections revealed histological alterations in the kidney tissues of adenine-induced CKD mice. Compared with the Normal group, the CKD group exhibited structural abnormalities, whereas these alterations appeared to be attenuated in the AP and HY7718-treaed groups.

### 2.2. Effect of HY7718 on Inflammatory Gene Expression in Colon Tissue

**Figure 2:**
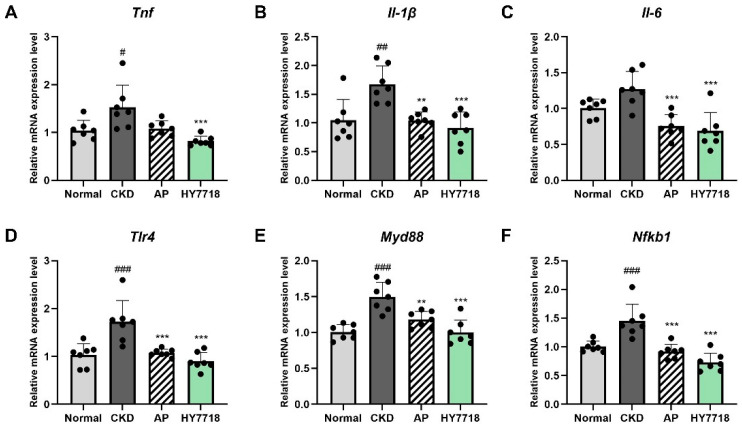
Effect of HY7718 on colonic inflammation-related gene expression in adenine-induced CKD mice. Relative mRNA expression levels of pro-inflammatory cytokine-related genes (**A**) *Tnf*, (**B**) Il-1β, and (**C**) *Il-6* as well as TLR4/MyD88/NF-κB signaling pathway-related genes (**D**) *Tlr4*, (**E**) *Myd88*, and (**F**) *Nfκb1* in colon tissues. Data are presented as mean ± standard deviation. Statistical significance was determined by one-way ANOVA followed by Tukey’s post hoc test. ^#^ *p* < 0.05, ^##^ *p* < 0.01, and ^###^ *p* < 0.001, compared with the Normal group; ** *p* < 0.01, and *** *p* < 0.001, compared with the CKD group. *Tnf*, Tumor necrosis factor-alpha; *Il-1β*; interleukin-1 beta; *Il-6*, interleukin-6; *Tlr4*, Toll-like receptor 4; *Myd88*, Myeloid Differentiation Primary Response 88; *Nfκb1*, nuclear factor kappa B subunit 1; CKD, adenine-induced CKD group; AP, allopurinol; HY7718, *L. plantarum* HY7718.

To investigate the effect of HY7718 on intestinal inflammatory responses, the expression levels of pro-inflammatory cytokines (*Tnf*, *Il-1β*, and *Il-6*) and TLR4/MyD88/NF-κB signaling-related genes (*Tlr4*, *Myd88*, and *Nfκb1*) were analyzed in colon tissues. As shown in Figure 2A–C, the CKD group exhibited elevated expression levels of *Tnf*, *Il-1β*, and *Il-6* compared with those in the Normal group. The increases in *Tnf* and *Il-1β* were statistically significant (*Tnf*, *p* < 0.05 and *Il-1β*, *p* < 0.01), whereas *Il-6* showed an increasing trend without reaching statistical significance. Administration of HY7718 significantly decreased the expression levels of these genes relative to those in the CKD group (*p* < 0.001). Treatment with AP also reduced the expression levels of *Il-1β* and *Il-6*, whereas *Tnf* expression was not significantly altered.

As illustrated in Figure 2D–F, a similar pattern was observed for genes involved in the TLR4/MyD88/NF-κB signaling pathway (*Tlr4*, *Myd88*, and *Nfκb1*). These genes showed significantly higher expression levels in the CKD group compared with those in the Normal group (*p* < 0.001), suggesting increased expression of genes associated with inflammatory signaling. HY7718 intake significantly downregulated the expression levels of *Tlr4*, *Myd88*, and *Nfκb1* (*p* < 0.001) relative to those in the CKD group. AP treatment also significantly reduced the expression levels of these genes (*Tlr4* and *Nfκb1*, *p* < 0.001; *Myd88*, *p* < 0.01). Collectively, intestinal inflammatory gene expression levels tended to be lower in the HY7718 group than in the AP group.

### 2.3. Effect of HY7718 on Gastric Motility-Related Gene Expression in Stomach Tissues

**Figure 3:**
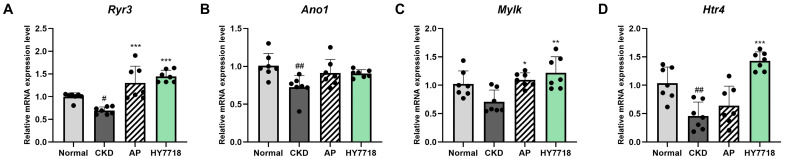
Effect of HY7718 on gastric motility-related gene expression in the stomach tissues of adenine-induced CKD mice. Relative mRNA expression levels of gastric motility-related genes (**A**) *Ryr3*, (**B**) *Ano1*, (**C**) *Mylk*, and (**D**) *Htr4* in stomach tissues. Data are presented as the mean ± standard deviation. Statistical significance was determined by one-way ANOVA followed by Tukey’s post hoc test. ^#^ *p* < 0.05, and ^##^ *p* < 0.01, compared with the Normal group; * *p* < 0.05, ** *p* < 0.01, and *** *p* < 0.001, compared with the CKD group. *Ryr3*, Ryanodine receptor 3; *Ano1*, Anoctamin-1; *Mylk*, Myosin light chain kinase; *Htr4*, 5-hydroxytryptamine receptor 4; CKD, adenine-induced CKD group; AP, allopurinol; HY7718, *L. plantarum* HY7718.

To assess the potential effect of HY7718 on gastrointestinal motility, the expression levels of gastric motility-related genes (*Ryr3*, *Ano1*, *Mylk*, and *Htr4*) were examined in stomach tissues (Figure 3). The CKD group exhibited generally lower expression levels of these genes than those in the Normal group. Among them, *Ryr3* (*p* < 0.01), *Ano1* (*p* < 0.01), and *Htr4* (*p* < 0.01) showed significantly lower expression levels compared with those in the Normal group, whereas *Mylk* showed a decreasing tendency but no statistical significance. HY7718 administration increased the expression levels of all motility-related genes compared with those in the CKD group. In particular, *Ryr3* (*p* < 0.001), *Mylk* (*p* < 0.01), and *Htr4* (*p* < 0.001) expression levels were significantly elevated following HY7718 supplementation. Treatment with AP also increased the expression of these genes relative to that in the CKD group; however, significant differences were observed only for *Ryr3* (*p* < 0.001) and *Mylk* (*p* < 0.05). Although *Htr4* expression increased after AP treatment, the magnitude of change was minimal. Further, *Ano1* expression levels tended to increase following both HY7718 and AP administration compared with those in the CKD group, but these differences were not statistically significant.

### 2.4. Effect of HY7718 on Digestive Enzyme Activity in Small Intestine Tissue

**Figure 4:**
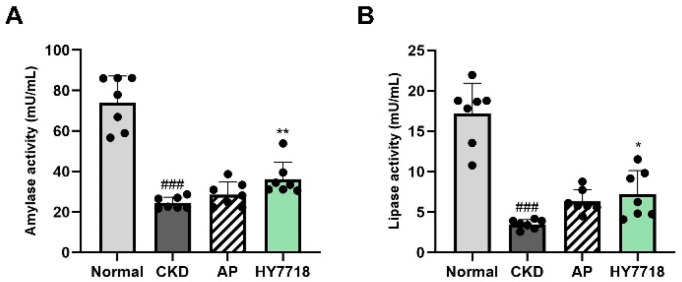
Effect of HY7718 on digestive enzyme activity in the small intestine tissue of adenine-induced CKD mice. (**A**) Amylase activity, and (**B**) Lipase activity in small intestine tissues. Data are presented as mean ± standard deviation. Statistical significance was determined using one-way ANOVA followed by Tukey’s post hoc test. ^###^ *p* < 0.001, compared with the Normal group; * *p* < 0.05 and ** *p* < 0.01, compared with the CKD group. CKD, adenine-induced CKD group; AP, allopurinol; HY7718, *L. plantarum* HY7718.

To evaluate whether HY7718 affects digestive function under CKD conditions, the activities of digestive enzymes (amylase and lipase) were measured in small intestine tissues (Figure 4). Both amylase and lipase activities were significantly decreased in the CKD group than in the Normal group (*p* < 0.001), suggesting impaired digestive enzyme function in CKD mice. HY7718 supplementation significantly increased the activities of both enzymes compared with those in the CKD group (Amylase, *p* < 0.01 and lipase, *p* < 0.05). AP treatment also elevated the digestive enzyme activities; however, amylase and lipase activities did not reach statistical significance. Despite these improvements, the activities of both enzymes remained lower than those in the Normal group.

### 2.5. Effect of HY7718 on Gut Microbiota Diversity and Composition

**Figure 5:**
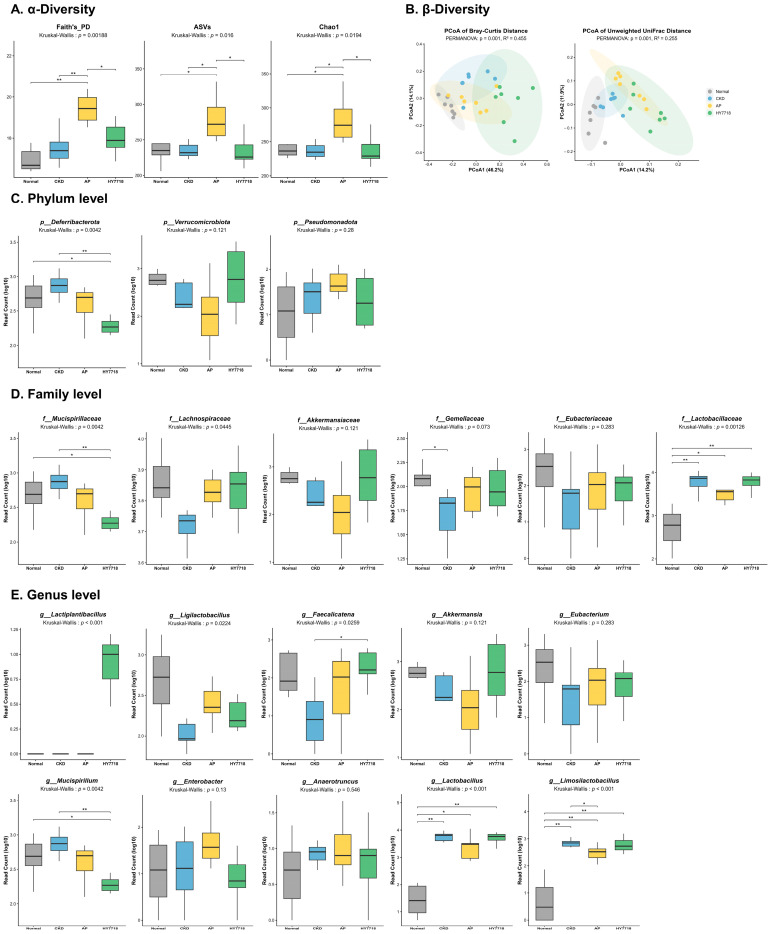
Gut microbiota in the mouse model of adenine-induced CKD. (**A**) α-diversity, (**B**) Principal coordinate analysis (PCoA) of β-diversity, (**C**) Taxonomy abundance at the phylum level, (**D**) Taxonomy abundance at the family level, and (**E**) Taxonomy abundance at the genus level. In the graph, gray represents the Normal group, blue represents the CKD group, yellow represents the AP group, green represents HY7718 group. Statistical significance was determined by Kruskal–Wallis (* *p* < 0.05, and ** *p* < 0.01). CKD, adenine-induced CKD group; AP, allopurinol; HY7718, *L. plantarum* HY7718.

To investigate whether HY7718 supplementation influences the gut microbial community under CKD conditions, 16S rRNA gene sequencing was performed using cecal samples from experimental mice. Alpha diversity was assessed using Faith’s phylogenetic diversity (PD), observed amplicon sequence variants (ASVs), and Chao1 indices (Figure 5A). Significant differences were detected among groups (Faith’s PD, *p* = 0.00188; ASVs, *p* = 0.016; Chao1, *p* = 0.0194). The AP group presented the highest alpha diversity across all indices, whereas no substantial differences were observed among the Normal, CKD, and HY7718 groups. Beta diversity was assessed using Bray–Curtis and unweighted UniFrac distance metrics, followed by principal coordinate analysis (PCoA) (Figure 5B). PERMANOVA confirmed significant differences in the microbial community structure among groups (*p* = 0.001). The CKD group showed a distinct microbial profile compared with that in the Normal group, whereas the HY7718 group showed a shifted community structure relative to that in the CKD group.

At the phylum level, the abundance of Deferribacterota differed significantly among groups (*p* = 0.0042), showing an increase in the CKD group and a reduction following HY7718 supplementation (Figure 5C). In contrast, the relative abundance of Verrucomicrobiota and Pseudomonadota showed no differences among the experimental groups. However, Verrucomicrobiota showed a decreasing trend in the CKD group and a relative increase following HY7718 supplementation, whereas Pseudomonadota tended to increase in the CKD and AP groups. At the family level, significant differences among groups were observed for *Mucispirillaceae* (*p* = 0.0042), *Lachnospiraceae* (*p* = 0.0445), and *Lactobacillaceae* (*p* = 0.00126) (Figure 5D). The abundance of *Mucispirillaceae* was increased in the CKD group and reduced following HY7718 supplementation. In contrast, *Lachnospiraceae* showed a lower abundance in the CKD group, whereas the AP and HY7718 groups exhibited relatively higher levels. *Lactobacillaceae* was markedly enriched in the CKD group and remained elevated in the HY7718 group. No significant differences were observed for *Akkermansiaceae* and *Gemellaceae*; however, both taxa tended to decrease in the CKD group and increase following HY7718 supplementation, whereas *Enterobacteriaceae* showed a contrasting tendency. *Eubacteriaceae* did not exhibit notable differences among groups. At the genus level, several taxa showed differential abundance patterns across the experimental groups (Figure 5E). Genera belonging to the *Lactobacillaceae* family showed distinct responses. *Lactobacillus* and *Limosilactobacillus* were significantly enriched in the CKD group and remained abundant following HY7718 supplementation (*p* < 0.001). In contrast, *Ligilactobacillus* was reduced in the CKD group and increased in both the AP and HY7718 groups (*p* = 0.0224). Notably, *Lactiplantibacillus* was detected almost exclusively in the HY7718 group (*p* < 0.001). Further, *Faecalicatena* was reduced in the CKD group and increased in the AP and HY7718 groups (*p* = 0.0259), whereas *Mucispirillum* showed the opposite pattern, with an increased abundance in the CKD group and reduced abundance following HY7718 supplementation (*p* = 0.0042). *Akkermansia*, *Eubacterium*, *Enterobacter*, and *Anaerotruncus* did not differ significantly among groups, although *Akkermansia* and *Eubacterium* tended to decrease in the CKD group and increase following HY7718 treatment, whereas *Enterobacter* and *Anaerotruncus* showed the opposite tendency.

**Figure 6:**
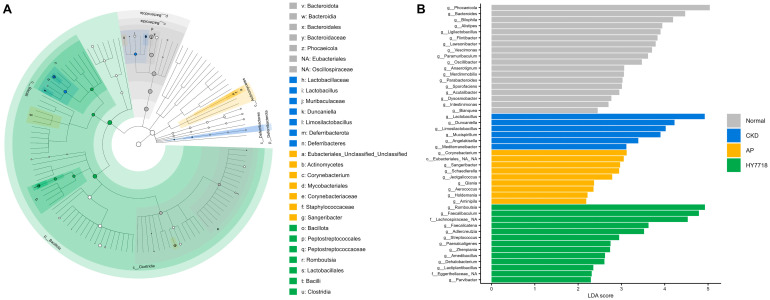
LEfSe analysis of gut microbiota composition. (**A**) Cladogram illustrating the phylogenetic distribution of discriminative taxa across taxonomic levels. (**B**) LDA score bar plot showing differentially abundant taxa with an LDA score > 2.0 and *p* < 0.05. Taxa labels were omitted from the cladogram for clarity.

As showed in Figure 6, LEfSe analysis identified a total of 46 taxa that were significantly different among the Normal, CKD, AP, and HY7718 groups (LDA score > 2.0, *p* < 0.05). The LDA score bar plot revealed distinct microbial signatures associated with each group. The cladogram further illustrated the phylogenetic distribution of these discriminative taxa across multiple taxonomic levels.

### 2.6. Correlation Analysis Between Host Phenotypes and Gut Microbial Taxa

**Figure 7:**
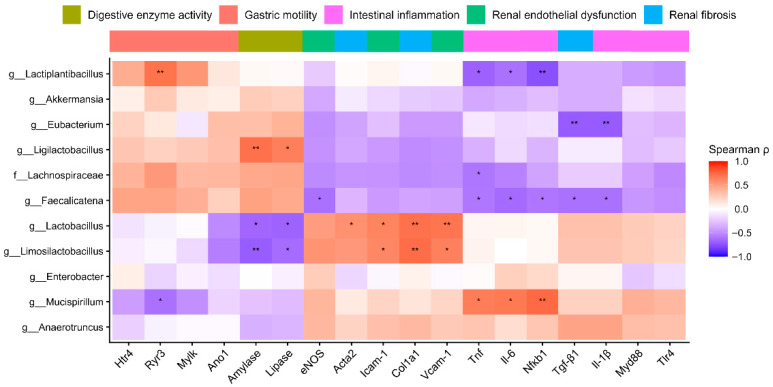
Correlation heatmap of gut microbial taxa and host phenotypic parameters. The heatmap was generated using Spearman’s rank correlation analysis. Phenotypic parameters were annotated into functional categories including digestive enzyme activity, gastric motility, intestinal inflammation, renal endothelial dysfunction, and renal fibrosis. Positive correlations are represented in red, and negative correlations are represented in blue, with the intensity indicating the strength of the correlation. *p*-values were adjusted for multiple comparisons using the Benjamini–Hochberg false discovery rate method. Statistical significance is indicated as follows: * *p* < 0.05 and ** *p* < 0.01.

To explore the relationships between the gut microbial taxa and host phenotypic parameters, Spearman’s correlation analysis was performed and the results were visualized as a heatmap (Figure 7). Overall, distinct correlation patterns were observed between microbial taxa and markers of renal, intestinal, and gastrointestinal function. Significant correlations were observed between specific microbial taxa and various CKD-related markers. *Lactiplantibacillus* was positively correlated with gastric motility genes (*Htr4*, *Ryr3*, *Mylk*, *Ano1*) and digestive enzyme activity (amylase and lipase), but negatively correlated with pro-inflammatory cytokines (*Tnf*, *Il-6* and *Nfκb1*) and renal fibrosis markers (*Tgf-β1* and *Acta2*). *Ligilactobacillusctobacillus* showed significant positive correlations with digestive enzyme activity. The abundance of *Lachnospiraceae*, *Faecalicatena*, and *Eubacterium* showed significant negative correlations with the intestinal inflammation and renal fibrosis markers. In contrast, *Mucispirillum* was positively correlated with genes related to intestinal inflammation (*Tnf*, *Il-6*, and *Nfκb1*) and negatively associated with gastric motility-related genes and digestive enzyme activity. *Lactobacillus* and *Limosilactobacillus* showed significant negative correlations with digestive enzyme activity, but positive correlations with renal fibrosis- and endothelial dysfunction-related genes.

## 3. Discussion

Chronic kidney disease (CKD) is a progressive disorder characterized by the gradual decline of renal function and remains a major global health burden [20]. Increasing evidence has highlighted the importance of the gut–kidney axis in CKD progression, suggesting that modulating intestinal homeostasis may represent a potential therapeutic strategy [6]. The present study examined whether supplementation with *Lactiplantibacillus plantarum* HY7718 modulates gut–kidney axis-associated alterations in adenine-induced CKD mice. HY7718 supplementation was associated with reduced expression of genes related to renal fibrosis and endothelial dysfunction, decreased expression of inflammation-related genes in the colon, and changes in gastrointestinal function-related parameters, including gastric motility-related gene expression and digestive enzyme activity. Taken together, these findings suggest that HY7718 may influence CKD-associated renal, intestinal, and gastrointestinal alterations, potentially through the gut–kidney axis.

Fibrosis is a pathological response to the chronic tissue injury characterized by excessive deposition of extracellular matrix components, which leads to structural remodeling and progressive organ dysfunction [21]. Renal fibrosis is a common pathological feature driving CKD progression and represents a major determinant of irreversible renal damage. In adenine-induced CKD models, tubular injury caused by persistent deposition of 2,8-dihydroxyadenine (2,8-DHA) crystals promotes tubulointerstitial fibrosis development. This process is largely mediated by profibrotic signaling pathways, particularly transforming growth factor-β1 (TGF-β1) [22]. Activation of TGF-β1 signaling pathways stimulates excessive deposition of extracellular matrix (ECM) components and myofibroblast differentiation. These processes are reflected by increased expression of fibrosis-related markers such as *Col1a1* and *Acta2*, which ultimately lead to tubulointerstitial fibrosis and progressive loss of renal function [21,23].

Furthermore, vascular inflammation and endothelial dysfunction have been increasingly recognized as important contributors to renal injury in CKD. Under physiological conditions, endothelial cells form a protective barrier lining the vasculature and maintaining vascular integrity and homeostasis [24]. However, in CKD, systemic inflammation, oxidative stress, uremic toxins, and metabolic risk factors such as hypertension, diabetes, and dyslipidemia can disrupt endothelial structure and function throughout the vascular system [25,26]. This endothelial dysfunction is often accompanied by endothelial activation, which is characterized by elevated expression of adhesion molecules such as ICAM-1 and VCAM-1 [27]. Our findings indicate that adenine-induced CKD mice showed increased expression of genes related to fibrosis and endothelial dysfunction, suggesting activation of profibrotic and inflammatory pathways in renal tissues. HY7718 administration significantly decreased the expression of these markers. Collectively, these findings suggest that HY7718 may be associated with modulation of gene expression related to fibrotic and inflammatory pathways in the kidney.

Accumulating evidence indicates that intestinal inflammation plays an important role in CKD progression through the gut–kidney axis. Notably, renal dysfunction can disrupt intestinal homeostasis, thereby promoting inflammatory responses in the gut and aggravating systemic inflammation and renal injury [7,28]. Activation of the TLR4/MyD88/NF-κB signaling pathway has been recognized as a key regulator of intestinal inflammatory responses because it induces the production of pro-inflammatory cytokines, including TNF-α, IL-1β, and IL-6 [29,30]. Consistent with these observations, the adenine-induced CKD mice in this study showed increased expression of pro-inflammatory cytokines and TLR4/MyD88/NF-κB signaling-related genes in colonic tissues. Administration of HY7718 significantly decreased the expression of these genes, suggesting that HY7718 may influence intestinal inflammatory signaling pathways within the gut–kidney axis.

Gastrointestinal dysfunction has increasingly been recognized in patients with CKD and may contribute to disturbances in intestinal homeostasis [12]. Clinical studies have reported that patients with CKD frequently experience gastrointestinal symptoms such as dyspepsia, nausea, and delayed gastric emptying, suggesting impaired gastrointestinal motility [12,13,31]. Disturbed gastric motility may disrupt coordinated gastrointestinal smooth muscle activity and alter the normal transit of luminal contents [14,32]. Our results indicated that adenine-induced CKD mice demonstrated reduced expression of gastric motility-related genes, including *Ryr3*, *Ano1*, *Mylk*, and *Htr4*, indicating impaired regulation of gastric smooth muscle activity. HY7718 supplementation increased the expression of these genes, suggesting potential modulation of gastric motility-related pathways. Furthermore, CKD mice showed decreased activities of digestive enzymes such as amylase and lipase in the small intestine, whereas HY7718 administration partially increased these enzyme activities compared with CKD mice. These findings suggest that HY7718 may be associated with modulation of gastrointestinal function-related parameters under CKD conditions.

In the present study, we explored the role of gut microbiota in CKD progression and the potential therapeutic effects of HY7718 supplementation. The results of our correlation analysis between gut microbial taxa and host phenotypic parameters revealed significant associations with renal, intestinal, and gastrointestinal markers, supporting a possible role of the gut–kidney axis in CKD pathophysiology. Deferribacterota showed a significant increase in the CKD group, which was subsequently reduced following HY7718 supplementation. This is consistent with prior studies indicating that Deferribacterota is associated with inflammatory responses and gut dysbiosis [33]. Pseudomonadota, which is linked to inflammation and dysbiosis in various pathological conditions, including CKD, exhibited a trend of increased abundance in the CKD group compared with that in the Normal group, although this change was not statistically significant [34]. *Mucispirillaceae* and *Mucispirillum* exhibited distinct patterns in response to CKD progression and HY7718 supplementation. *Mucispirillum* was significantly enriched in the CKD group, and supplementation with HY7718 led to a reduction in its abundance. This taxon, commonly associated with intestinal inflammation and dysbiosis, has been implicated in regulating gut microbial diversity and influencing host immune responses [35]. Increased *Mucispirillum* in the CKD group suggests that it may play a role in promoting gut inflammation, a common feature of CKD. Interestingly, after HY7718 supplementation, Deferribacterota, Pseudomonadota, and *Muribaculaceae*/*Mucispirillum* abundance showed a decreasing trend, suggesting a potential association between HY7718 supplementation and microbiota changes related to inflammatory conditions. This observation aligns with the notion that probiotic supplementation can influence gut microbial composition and be associated with inflammatory status. In particular, *Mucispirillum* was positively correlated with markers of intestinal inflammation, such as *Tnf*, *Il-6*, and *Nfκb1*, consistent with previous reports linking this taxon to inflammatory conditions [29,30]. Notably, the abundance of *Mucispirillum* decreased following HY7718 supplementation, suggesting that HY7718 supplementation may be associated with changes in gut microbial composition and intestinal inflammation-related markers. These findings suggest that HY7718 supplementation may be associated with shifts in gut microbial composition, including taxa previously linked to inflammatory conditions, potentially contributing to gut–kidney axis-related processes. In the present study, the relative abundances of *Lachnospiraceae*, *Faecalicatena*, and *Eubacterium* were significantly altered in the CKD group and following HY7718 supplementation, suggesting an association of these taxa with intestinal inflammation and CKD-related processes. These genera are well-known for their involvement in the production of short-chain fatty acids (SCFAs), such as acetate, butyrate, and propionate, which play crucial roles in maintaining gut health and modulating systemic inflammation [36,37,38]. *Lachnospiraceae* and *Faecalicatena* were significantly reduced in the CKD group but increased following HY7718 supplementation. *Eubacterium* showed no significant difference but exhibited a similar trend. The reduction in these taxa in the CKD group may suggest an alteration in SCFA-related metabolic pathways, which has been associated with intestinal inflammation in previous studies The increase in these taxa following HY7718 supplementation may be associated with enrichment of SCFA-producing bacteria, which may be associated with microbial features linked to gut homeostasis and inflammatory status. In addition to modulating microbial abundance, our analysis revealed that certain taxa, including *Lachnospiraceae*, *Faecalicatena*, and *Eubacterium*, showed significant negative correlations with markers of intestinal inflammation and renal fibrosis, consistent with previous reports linking these taxa to intestinal barrier-related functions. Changes in the abundance of these taxa following HY7718 supplementation may be associated with shifts in microbiota linked to inflammatory and fibrotic markers. We found that Verrucomicrobiota, *Akkermansiaceae*, and *Akkermansia* exhibited a decreasing trend in the CKD group. Notably, after HY7718 supplementation, the abundance of these taxa showed a tendency toward the levels observed in the Normal group. *Akkermansia*, known for its role in maintaining intestinal barrier function and modulating inflammation, showed a tendency to recover following HY7718 supplementation, suggesting a potential beneficial effect in alleviating intestinal inflammation and CKD progression [39]. Despite the lack of statistical significance, the observed shift in these microbial taxa further supports the idea that HY7718 supplementation may be associated with changes in microbial composition linked to CKD-related alterations. In this study, *Lactiplantibacillus*, a key component of the HY7718 strain, was predominantly detected in the HY7718 supplementation group, suggesting enrichment of this genus following HY7718 supplementation. This genus has been reported to be associated with probiotic functions in previous studies, including intestinal inflammation modulation, gut barrier strengthening, and gastrointestinal motility regulation [18]. The high abundance of *Lactiplantibacillus* in the HY7718 group, compared with the minimal detection in the CKD and Normal groups, supports the idea that HY7718 supplementation may be associated with changes in gut microbial composition. Furthermore, correlation analysis suggested that *Lactiplantibacillus* was associated with inflammatory processes and gastrointestinal function-related parameters in CKD.

Interestingly, *Lactobacillus* and *Limosilactobacillus* exhibited significantly increased abundance in the CKD group compared with the Normal group. Members of the genus *Lactobacillus* have been reported to be associated with intestinal homeostasis and inflammatory responses in previous studies [40]. However, previous studies have shown that specific *Lactobacillus* species were significantly enriched in adenine-induced CKD models [41]. The increased abundance of these genera in the CKD group may be associated with CKD-related alterations in the gut microbial environment. Furthermore, the abundance of *Lactobacillus* and *Limosilactobacillus* remained elevated in the HY7718 supplementation group, which may be associated with ecological interactions within the altered gut microbial community.

In this study, we demonstrated that *Lactiplantibacillus plantarum* HY7718 supplementation was associated with changes in gene expression related to intestinal inflammation, renal fibrosis, and gastrointestinal function in an adenine-induced CKD mouse model, potentially through the gut–kidney axis. HY7718 supplementation was associated with reduced expression of fibrosis- and inflammation-related genes, as well as alterations in gut microbiota composition. Future studies should focus on conducting metabolomic analyses to better characterize microbiota-derived metabolic changes. In addition, further investigation is warranted to identify the bioactive components of HY7718, including surface layer proteins (SLPs), extracellular vesicles, and exopolysaccharides (EPS), and to elucidate their roles in modulating host inflammatory and fibrotic pathways. Finally, well-designed clinical studies will be required to determine the translational relevance of these findings and to evaluate the potential of HY7718 as a functional intervention in CKD.

## 4. Materials and Methods

### 4.1. Preparation of Lactiplantibacillus plantarum HY7718

*Lactiplantibacillus plantarum* HY7718 (HY7718), originally isolated from a traditional Korean fermented squid food (squid jeotgal), was provided by hy Co., Ltd. (Yongin-si, Republic of Korea). The bacterial strain in MRS broth (BD Difco, Sparks, MD, USA) with 20% (*v*/*v*) glycerol (Sigma-Aldrich, St. Louis, MO, USA) was preserved at −80 °C. HY7718 was cultured anaerobically at 37 °C for 24 h in MRS broth. For use in animal experiments, freshly cultured HY7718 was freeze-dried and administered to the animals via oral gavage.

### 4.2. Animals and Experimental Design

Eight-week-old male C57BL/6 mice (Dooyeol Biotech, Seoul, Republic of Korea) were housed in standard laboratory cages under controlled environmental conditions (50 ± 10% relative humidity, 23 ± 2 °C temperature, and a 12 h light/dark cycle). After a 7-day acclimatization period, the mice were randomly assigned to four groups (*n* = 7 per group) for the duration of the experiment: Normal group (Normal), fed an AIN-93G diet; the Chronic Kidney Disease-induced (CKD) group, induced with a 0.15% adenine-containing AIN-93G diet; the Allopurinol (AP, the positive control) group, which was administered allopurinol at 20 mg/kg/day by oral gavage and induced with the adenine diet; and the *L. plantarum* HY7718 (HY7718) group, which was administered HY7718 at 10^8^ CFU/kg/day of HY7718 by oral gavage and induced with the adenine diet. Treatments were administered daily for a total of 7 weeks. For the first 4 weeks, the Normal and CKD groups were administered saline by oral gavage, whereas the AP and HY7718 groups were treated with allopurinol at 20 mg/kg/day and HY7718 at 10^8^ CFU/kg/day, respectively, by oral gavage. After this initial period, the CKD, AP, and HY7718 groups continued their respective treatments for an additional 3 weeks, during which they were also maintained on a 0.15% adenine-supplemented diet to induce CKD.

### 4.3. Tissue Collection and Sample Preparation

At the end of the experiment, all animals were euthanized by CO_2_ asphyxiation. Kidney, stomach, small intestine, colon, and cecum tissues were harvested and immediately stored at −80 °C for subsequent analyses. All procedures were conducted in accordance with the ethical guidelines for animal research and were approved by the Ethics Review Committee of R&BD Center, hy Co., Ltd., Republic of Korea (Approval No. AEC-2025-0003-Y).

### 4.4. Histological Analysis of Kidney Tissues

Kidney tissues were fixed in 10% neutral-buffered formalin, embedded in paraffin, and sectioned at 4–5 μm thickness. The sections were stained with hematoxylin and eosin (H&E) using standard procedures and examined under a light microscope (Dooyeol Biotech). Images were captured using MoticDSAssistant (Motic VM V1 Viewer 2.0).

### 4.5. Gene Expression Analysis Using Quantitative Real-Time PCR

Total RNA was extracted from the kidney, stomach, and colon tissues using the Easy-spin Total RNA Extraction Kit (iNtRON Biotechnology, Seoul, Republic of Korea) following the manufacturer’s instructions. RNA quality and quantity were assessed using an Agilent BioTek Gen5 (Agilent Technologies, Santa Clara, CA, USA). cDNA synthesis was carried out using the Omniscript Reverse Transcription Kit (Qiagen, Hilden, Germany) at 37 °C for 1 h.

Quantitative real-time PCR was performed using the TaqMan^TM^ Gene Expression Master Mix (Applied Biosystems, Waltham, MA, USA) on a QuantStudio 6 Flex Real-time PCR System (Applied Biosystems). The PCR conditions were as follows: initial denaturation at 95 °C for 10 min, followed by 45 cycles of denaturation at 95 °C for 15 s and annealing/extension at 60 °C for 1 min. Gene-specific primers were used for target genes and *Gapdh* was used as a housekeeping gene for normalization. Relative gene expression was calculated using the 2^−ΔΔCt^ method. The list of target genes used in this experiment is provided in Table 1.

### 4.6. Measurement of Digestive Enzyme Activity

Amylase, lipase, and trypsin activities in the small intestine tissues were measured using the Amylase Assay Kit (ab102523), Lipase Activity Assay Kit (ab102524), and Trypsin Activity Assay Kit (ab102531), respectively, according to the manufacturer’s protocols (Abcam, Cambridge, UK). Enzyme activities were determined by measuring absorbance at the appropriate wavelengths on a BioTek^®^ Synergy HT Microplate Reader (Agilent Technologies, Santa Clara, CA, USA). Kinetic measurements were performed to assess enzyme activity over time, and the results were calculated based on changes in absorbance.

### 4.7. Genomic DNA Extraction and Quantification

Genomic DNA was extracted from cecal tissue using the QIAmap Fast DNA Stool Mini Kit (Qiagen, Hilden, Germany), following the manufacturer’s protocol. The extracted genomic DNA (gDNA) concentration was quantified using the Quant-iT™ PicoGreen^®^ dsDNA Assay Kit (Invitrogen, Waltham, MA, USA), as per the manufacturer’s instructions.

### 4.8. 16S rRNA Amplicon and Library

The V3–V4 regions of the bacterial 16S rRNA gene were amplified using the Illumina 16S metagenomic workflow. PCR was performed using 5 ng of purified genomic DNA, Hercules II Fusion DNA Polymerase (Agilent Technologies, Santa Clara, CA, USA), 1 mM dNTPs, and 500 nM primers. The PCR conditions included an initial denaturation at 95 °C for 3 min, followed by 25 cycles of 95 °C for 30 s, 55 °C for 30 s, and 72 °C for 30 s, with a final extension at 72 °C for 5 min. The primers used for amplification were designed to include Illumina adapter overhangs, with the sequences V3-F (5′-TCGTCGGCAGCGTCAGATGTGTATAAGAGACAGCCTACGGGNGGCWGCAG-3′) and V4-R (5′-GTCTCGTGGGCTCGGAGATGTGTATAAGAGACAGGACTACHVGGGTATCTAATCC-3′).

The PCR products were purified using AMPure XP beads (Beckman Coulter, Brea, CA, USA), and indexed using the Nextera XT Index Kit (Illumina, San Diego, CA, USA) with 10 cycles of amplification. The indexed libraries were purified and quantified using the KAPA Library Quantification Kit (KAPA Biosystems, Wilmington, MA, USA) and the Agilent D1000 ScreenTape system (Agilent Technologies, Santa Clara, CA, USA). Sequencing was conducted in the paired-end mode (2 × 300 bp) using the MiSeq™ platform (Illumina) at Macrogen (Seoul, Republic of Korea).

### 4.9. Raw Data Processing and Quality Control

Illumina MiSeq raw data were processed by demultiplexing the samples using index sequences to generate paired-end FASTQ files. Adapter and primer sequences were removed using Cutadapt (v3.2), and the resulting forward (250 bp) and reverse (200 bp) reads were retained for further analyses. Error correction was performed using DADA2 (v1.18.0) in R (v4.0.3), excluding reads with an expected error rate > 2. Chimeric sequences were discarded, and Amplicon Sequence Variants (ASVs) were generated.

ASVs shorter than 350 bp were excluded. For microbiome analysis, QIIME (v1.9) was used for data normalization via subsampling, based on the sample with the fewest reads. Taxonomy was assigned by aligning the ASVs with the NCBI 16S Microbial Database using BLAST+ (v2.9.0), with a minimum query coverage of 85% and an identity of 85% for valid matches.

### 4.10. Microbial Community Analysis

Microbial community composition was analyzed using QIIME2 based on the ASV abundances and taxonomy information. Alpha diversity was assessed by calculating the Faith’s PD, Observed ASVs and Chao1. These indices were used to evaluate the species diversity and evenness within each sample. Beta diversity was analyzed using Bray–Curtis and Unweighted UniFrac distances to evaluate the differences in microbial community composition between samples. Principal Coordinate Analysis (PCoA) was performed to visualize the relationships among samples based on Beta diversity metrics. Statistical differences in microbial community composition across groups were assessed using permutational multivariate analysis of variance (PERMANOVA).

Differentially abundant taxa among groups were identified using linear discriminant analysis effect size (LEfSe) implemented in the microbiomeMarker package in R. The analysis was performed using genus-level aggregated data. Statistical significance was determined using the Kruskal–Wallis test followed by the Wilcoxon rank-sum test, and taxa with a linear discriminant analysis (LDA) score > 2.0 and *p* < 0.05 were considered significantly discriminative features. LDA score bar plots were generated to visualize the effect size of differentially abundant taxa. For cladogram visualization, LEfSe analysis was performed using all taxonomic levels to illustrate the phylogenetic distribution of discriminative taxa.

All next-generation sequencing datasets generated in this study have been deposited in the NCBI Sequence Read Archive (SRA) under accession code (PRJNA1453691).

### 4.11. Correlation Heatmap

Spearman’s rank correlation analysis was conducted to assess the relationships between microbial taxon abundances and host phenotypic parameters. A correlation matrix was generated and visualized as a heatmap using the ggplot2 package in R (version 4.0.3). To correct for multiple comparisons across all taxon–phenotype pairs, *p*-values were adjusted using the Benjamini–Hochberg false discovery rate (FDR) method. Statistical significance was defined as FDR-adjusted *p* < 0.05.

### 4.12. Statistical Analysis

Animal experiment data were analyzed using GraphPad Prism 10 software (GraphPad Software, San Diego, CA, USA). Results are presented as the mean ± standard deviation (SD). Statistical comparisons between groups were performed using one-way ANOVA, followed by Tukey’s post hoc test for multiple comparisons. Bioinformatics data were evaluated using the Kruskal–Wallis test, followed by Dunn’s post hoc test for pairwise comparisons. p-values were adjusted for multiple comparisons using the Benjamini–Hochberg false discovery rate (FDR) method. A *p*-value less than 0.05 was defined as statistically significant.

## 5. Conclusions

The data presented in this study suggest that *Lactiplantibacillus plantarum* HY7718 has potential as a functional food supplement for modulating CKD-associated pathological changes. HY7718 supplementation was associated with changes in the expression of genes related to renal fibrosis, vascular inflammation, and intestinal inflammation. In addition, HY7718 was associated with alterations in gastrointestinal function-related gene expression and digestive enzyme activity, as well as shifts in gut microbiota composition.

Collectively, these findings suggest that HY7718 may influence gut–kidney axis-associated pathways; however, further studies, including protein-level validation and metabolite analyses, are required to confirm these effects and to elucidate the underlying mechanisms.

## Figures and Tables

**Table 1:** List of Taqman probes used in this study.

Gene Symbol	Gene Name	Assay ID
*Gapdh*	Glyceraldehyde-3-phosphate dehydrogenase	Mm99999915_g1
*Tgf-β1*	Transforming growth factor, beta 1	Mm01178820_m1
*Acta2*	Actin, alpha 1, skeletal muscle	Mm00808218_g1
*Col1a1*	Collagen, type I, alpha 1	Mm00801666_g1
*Icam-1*	Intercellular adhesion molecule 1	Mm00516023_m1
*Vcam-1*	Vascular cell adhesion molecule 1	Mm00449197_m1
*Tnf*	Tumor necrosis factor-alpha	Mm00443258_m1
*Il-1β*	Interleukin 1 beta	Mm00434228_m1
*Il-6*	Interleukin 6	Mm00446190_m1
*Tlr4*	Toll-like receptor 4	Mm00445273_m1
*Myd88*	Myeloid differentiation primary response gene 88	Mm00440338_m1
*Nfκb1*	Nuclear factor kappa B subunit 1	Mm00476361_m1
*Ryr3*	Ryanodine receptor 3	Mm01328421_m1
*Ano1*	Anoctamin 1, calcium activated chloride channel	Mm00724407_m1
*Mylk*	Myosin, light polypeptide kinase	Mm00653039_m1
*Htr4*	5 hydroxytryptamine (serotonin) receptor 4	Mm00434129_m1

## Data Availability

The data presented in this study are available upon request. 16S rRNA sequencing data have been deposited in the NCBI SRA under accession number PRJNA1453691.
